# Smoking Prevalence and Correlates among Chinese Immigrants: A Secondary Data Analysis Study

**DOI:** 10.3390/ijerph20085559

**Published:** 2023-04-18

**Authors:** Fang Lei, Eunice Lee, Joy Toyama

**Affiliations:** 1 School of Nursing, University of Minnesota Twin Cities, Minneapolis, MN 55455, USA; 2 School of Nursing, University of California Los Angeles, Los Angeles, CA 90095, USA

**Keywords:** Chinese immigrants, correlation, prevalence, secondary data analysis, smoking

## Abstract

Purpose: This study aimed to (1) identify the smoking prevalence among Chinese immigrants and (2) explore associations between their current smoking behaviors and demographic factors, psychological distress, and health utilization factors. Methods: Inclusion criteria were applied to extract data from the 2016 California Health Interview Survey; 650 eligible Chinese immigrant respondents were included in the sample. Independent variables were extracted based on the Integrated Model of Behavioral Prediction. Descriptive analyses and logistic regression were conducted using SAS 9.4 software. Results: 4.23% of the surveyed Chinese immigrants were current smokers. Chinese immigrants who were 50–65 years old, male, had less than a bachelor’s degree education level, and a lower income were more likely to be current smokers. Income was significantly associated with Chinese immigrants’ current smoking status (*p* = 0.0471). Conclusions: Chinese immigrants’ current smoking behaviors are significantly associated with their income. Interventions targeting low-income Chinese immigrants and tobacco price policies could potentially influence Chinese immigrants’ smoking behaviors. Health education about smoking cessation should focus on male Chinese immigrant smokers who are 50–65 years old and have less than a bachelor’s degree education and a lower income. More research needs to be carried out to encourage Chinese immigrants to quit smoking.

## 1. Introduction

### 1.1. Smoking Prevalence and Lung Cancer

Cigarette smoking is a significant problem in the US population. It is estimated that 480,000 people die from cigarette smoking every year in the United States [1]. Cigarette smoking is closely associated with cardiovascular diseases, shock, chronic respiratory diseases, and cancer [2]. It is a major cause of lung cancer, and it contributes to 87% of all lung cancer deaths among adults in the US [3]. Cigarette smoking-associated lung cancer is the second and fourth most common cancer for Chinese American men and women, respectively [4]. It is also the leading cause of cancer deaths among Chinese Americans [4]. It contributed to approximately 30% of all cancer-related deaths in Chinese Americans [4].

Chinese American is the largest Asian-American subgroup in the US, and they constitute 30% of the Asian American population [5]. In 2020, the total Asian population was 14,674,252 with a Chinese subset of 4,404,678, which included 46.5% male and 53.5% female [5]. Of the Chinese Americans, 671,110 were at or older than 65 years, 15.4% had less than a high school education level, and 6% were without any insurance [5]. While the smoking rate among the US population was 14% in 2019 [1], the smoking rate among Chinese Americans was 7.6% [6]. Even though the smoking rate for Chinese Americans is approximately half of that for the general US population, the relationship is mitigated by gender. After stratification, a significantly higher smoking rate was observed among Chinese American males compared to US males [7,8,9]. This relationship was reflected in previous studies. While Yu et al. [9]. reported that the smoking rate for Chinese American males was 34% (2% for females) in Chicago’s Chinatown, another study showed that the smoking rate for adult Chinese American males was 30.3% (2.2% for females) in New York City [7], which was approximately two-fold higher than that in the US male population (15.3–16.7%) [8].

### 1.2. Factors Associated with Smoking Behavior

Although evidence on the factors associated with Chinese immigrant smokers’ smoking behaviors is lacking, previous research has identified various factors that are related to Chinese Americans’ smoking behavior, including language proficiency [10], education level [9], social smoking norms [11], depression [12], acculturation [13] knowledge about smoking consequences [14] and perceived benefits of smoking cessation [15]. Higher English language proficiency was associated with decreased smoking rates among Chinese American men in a study conducted with 541 Chinese American adults [10]. In another study conducted with 644 Chinese Americans living in Chicago’s Chinatown, a low level of education (odds ratio (OR), 2.41; 95% confidence interval (CI), 1.31–4.46), use of a non-Western physician or clinic for health care (OR, 2.64; 95% CI, 1.46–4.80), and no knowledge of early cancer warning signs and symptoms (OR, 2.52; 95% CI, 1.35–4.70) were significantly associated with increased smoking rates among Chinese American men [9].

Within the Chinese American population, compared to people who have never smoked, current smokers were less likely to be proficient in English [10]. They had lower levels of knowledge about the health effects of tobacco and were more likely to have traditional Chinese cultural beliefs about tobacco use [11,14]. Compared to former smokers, current smokers were less likely to have a regular doctor [11,14]. Additionally, higher depressive symptoms (M, 20.4; 95% CI, 18.8–22.2), higher lifetime prevalence rates of major depressive disorders (30.3%; 95% CI, 24.0–37.2%) and dysthymia (11.6%; 95% CI, 7.5–16.9%) were reported to be more prevalent among Chinese American smokers compared to the overall Chinese American population [12]. Furthermore, it was reported that a negative relationship exists between years living in the US, use of English, and attitude toward smoking among overall Chinese immigrants [13]. However, for Chinese females, acculturated Chinese females held a more positive smoking attitude and were more likely to smoke than less acculturated Chinese females [13].

### 1.3. Theoretical Framework

**Figure 1:**
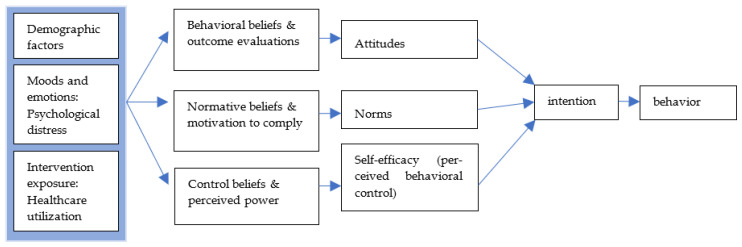
Integrated Model of Behavioral Prediction.

The Integrated Model of Behavioral Prediction was used to guide the study, especially for the measurement selection and analysis process. The Integrated Model of Behavioral Prediction [16] was developed based on Fishbein and Ajzen’s [17] reasoned action approach. As shown in Figure 1, the demographics, moods and emotions, and intervention exposure are among the distal variables that indirectly predict health behavior. Based on the Integrated Model of Behavioral Prediction, in this study, variables related to the demographic factors, psychological distress, and healthcare utilization were selected for further data analysis.

### 1.4. Study Purpose

Originating from another country, China, which has a higher smoking rate (33.8% in 2002 and 26.6% in 2022) [9,18] than the US, Chinese immigrants may encounter multiple challenges when adjusting to their new environment. The challenges may have both internal and external impact on Chinese immigrants’ smoking behaviors.

The purpose of this study was to (1) identify the smoking prevalence among Chinese immigrants and (2) explore the demographic factors, psychological distress, and healthcare utilization factors that are significantly associated with Chinese immigrants’ current smoking status. This study provides insight into Chinese immigrants’ current smoking behavior and its related factors, which may potentially inform tailored smoking cessation intervention programs that aim to decrease the smoking rate among Chinese immigrants.

## 2. Methods

### 2.1. Design and Ethical Approval

This is a secondary data analysis study. Data analyzed in this study were derived from the 2016 California Health Interview Survey (CHIS) Version 1 [19]. CHIS data were published on the UCLA Center for Health Policy Research website as a public data resource for facilitating public health research and medical development. Participants’ identifiable personal information was eliminated. This study was waived for the ethical approval (UCLA IRB#11-002227) since it was a secondary data analysis study, and no harm risk was induced for the participants.

### 2.2. Instrument

CHIS is the nation’s largest state-level health survey [19]. It is well-known for its hard-to-find data on special subgroups and provides relatively robust samples of major racial/ethnic groups, sexual minorities, and other populations living in California [19]. It was conducted in California on a continuous basis and covered a wide range of health topics [19]. In 2016, CHIS interviewed 21,269 households, including 21,055 adults, 840 teens and 2136 children [19]. Data for CHIS 2016 were collected by random-dialed telephone interviews [19]. To capture the rich diversity of the California population, interviews were conducted in six languages: English, Spanish, Vietnamese, Korean, Tagalog, and Chinese (Mandarin and Cantonese dialects) [19].

### 2.3. Data Quality

CHIS data were collected based on the approach of the Total Survey Error perspective [20], which is a data collection method addressing multiple threats to survey quality. Although sample selection bias related to the declining response rates was acknowledged, CHIS provided high-quality data that accurately represent the California household population [19].

### 2.4. Sample

The dataset that was analyzed from the CHIS 2016 data was the subset of individuals comprised of Chinese immigrants. Inclusion criteria for the participants were: (1) a person having origins in China [21] and (2) aged 18 years or older. Since participants in the CHIS survey were chosen by a random sampling method in the California population [19], the sample was considered extensive enough to be statistically representative [19] of California’s Chinese immigrant population. After applying the inclusion criteria, 650 Chinese immigrant respondents were included in the study, which was proven to be a sufficient sample size using power analysis by GPower software. To detect an odds ratio at 1.5, alpha error probability at 0.05, and power at 0.95, using logistic regression to detect differences will require a total sample size larger than 417 in this study.

### 2.5. Measurement Selection Process

The measurement selection process was conducted by the first author and triangulated by the second author. After reading the CHIS 2016 questionnaire item by item, the first author identified variables measuring demographic factors, psychological distress, and healthcare utilization factors from the CHIS 2016 data to make a table of evidence. Important data including dimension, variable, variable name, variable input, and question code were included in the analysis table. The table was checked by the second author and discussed further between the two authors to reach an agreement.

### 2.6. Independent Variables

In the study, independent variables related to demographic factors, psychological distress, and healthcare utilization factors were identified by the guidance of the theoretical framework and literature review results. Demographic variables included age, gender, marital status, spoken English use, level of English proficiency, years lived in the US, educational attainment, employment status, and income. Psychological distress related to the construct of moods and emotions included a variable measuring distress. Healthcare utilization variables related to the construct of intervention exposure (health care exposure) included the variables of having a personal doctor as a main medical provider and the language the doctor speaks. All the independent variables, except for income (ratio) and distress (interval), were categorical data.

### 2.7. Dependent Variable

The dependent variable chosen in this study was current smoking status. The dependent variable was categorical data.

### 2.8. Data Analysis

We used SAS 9.4 software to analyze data. A table including the dependent variable, independent variables, level of measurement, analysis method, results, and interpretation was generated to facilitate the data analysis process. Descriptive and logistic regression analyses were conducted. Multivariate models were established to check the correlation between independent and dependent variables. The *p*-value was set at the 0.05 level. Demographic characteristics were analyzed using weighted survey frequencies and weighted survey means. The survey methods utilized replicate weights and jackknife error estimates. Bivariate models were performed using survey weighted logistic regressions. When the outcomes were dichotomous, a logistic model was utilized, and when the outcome had more than two categories, a multinomial logistic regression was performed. The third author, who is also a statistician, performed the data analysis. The first and second authors checked the results and validated the data analysis process.

## 3. Results

### 3.1. Sample Characteristics

The results show that among the 650 Chinese immigrant respondents, 64.02% of them were aged 18–50 years old; 47.02% were male; 64.08% were married; and 59.42% spoke English well or very well. Most of the Chinese immigrant respondents (65.51%) lived in the United States for more than 15 years. Nearly half of the respondents (48.02%) had a bachelor’s degree or some graduate school level of education. More than half of them (69.2%) were employed, and 97.31% of them had a personal doctor as their main medical provider. More information on the demographic variables is provided in Table 1.

### 3.2. Current Smoking Status among Chinese Immigrants

The results show that 4.23% (95% CI, 0–9.29%) of the surveyed Chinese immigrants were current smokers. The prevalences of smoking among surveyed male and female Chinese immigrants were 5.95% and 2.70%, respectively. Among Chinese immigrants who were 18–50 years old, 0.53% were current smokers; among Chinese immigrants who were 50–65 years old, 15.17% were current smokers; and among Chinese immigrants who were 65+ years old, 4.02% were current smokers. Regarding the smoking prevalence among Chinese immigrants with different educational levels, the results show that 12.80% of Chinese immigrants who had less than a bachelor’s education were current smokers; 0.62% of Chinese immigrants who had BA/BS/some-graduate school education were current smokers; and 2% of Chinese immigrants who had MS/MA/doctorate education were current smokers. Among Chinese immigrants who were current smokers, the average income and median income in the last month were $221.51 (95% CI, −224.67–667.68), and $0 (95% CI, −5013.46–5013.46), respectively. Among Chinese immigrants who were not current smokers, the average income and median income in last month were $4339.55 (95% CI, 3007–5672.11), and $2566.28 (95% CI, −594.29–5726.84), respectively (Table 2A,B).

### 3.3. Factors Correlated with Current Smoking Status

As shown in Table 3, income was the only significant variable associated with current smoking status among Chinese immigrants. Chinese immigrant respondents who earned a lower income in the last month were more likely to be current smokers (*p* = 0.0471). The results show no significant associations among the variables measuring utilization of healthcare factors, psychological distress, other demographic factors, and the current smoking status. However, even though the *p* value was slightly higher than 0.05, age and employment status appear to be associated with current smoking status, at *p* = 0.0565 and *p* = 0.0943, respectively.

## 4. Discussion

Based on the CHIS 2016 data, this study explored the smoking prevalence and the correlations among variables measuring demographic factors, psychological distress, healthcare utilization, and the current smoking status among Chinese immigrants. Findings from this study could help to provide evidence about the smoking prevalence in different Chinese immigrant groups. Targeted smoking cessation programs could be designed by focusing on the Chinese immigrant groups with a high smoking prevalence. Necessary interventions could be implemented to decrease smoking rates among Chinese immigrant males aged between 50 and 65 years old with low-income and low-education levels.

In the study, the results show that the prevalence of cigarette smoking was low among surveyed Chinese immigrants in California in 2016. While the smoking rate was 4.23% among the whole surveyed Chinese immigrant population, the smoking rates were 5.95% and 2.70% among surveyed male and female Chinese immigrants, respectively. Compared to the smoking rate of 15.5% among the whole US population in 2016 and 7.6% in Chinese Americans in 2019 [6,22], the smoking rate was much lower in the Chinese immigrant population. Although this study only provided one cross-sectional view of the smoking rate among Chinese immigrants, the low smoking rate among the whole Chinese immigrant population (4.23%) cannot hide the fact of the high smoking rate in the 50 to 65-year-old group of Chinese immigrants (15.17%). Smoking cessation programs focusing on the 50 to 65-year-old age group of Chinese immigrants should be implemented, and further lung cancer screening programs should be introduced in this population.

In addition, the results from this study show that the current smoking rates were higher in surveyed male Chinese immigrants with less than a bachelor’s degree education and with a lower income, compared to the surveyed female Chinese immigrants with higher than a bachelor’s education and with a higher income. The high prevalence of current smoking behavior among surveyed male Chinese immigrants with less than a bachelor’s education and with a lower income may be related to the masculine culture element, lacking knowledge about the harm of smoking, and financial pressure [23,24]. Still, reasons for the high smoking rates among these population groups need to be further explored.

In the study, findings showed that the level of income was significantly associated with current smoking status among Chinese immigrants. This result is somewhat similar to the findings from a study on the Hispanic adult population who resided in a community in New Mexico [25]. The researchers examined whether household income, education, and language preference were predictors of cigarette smoking in the Hispanic adult population. Their results show that household income was one of the variables that predicted continued smoking among ever-smokers [25]. Similarly, another study conducted in Hungary [24] also showed that income was associated with large differences in smoking prevalence in the 25–64 age group of Hungarian citizens. The higher smoking prevalence among low-income Chinese immigrant smokers may relate to the decreased smoking cessation access and decreased availability of smoking cessation seminars, medicines, counseling, etc., or it could be related to the occupational smoking control policy varying from different occupations. Interventions such as price policies were suggested to prevent addiction to smoking among the lower-income population [26]. Culturally sensitive smoking cessation programs targeted at low-income Chinese immigrant smokers could also help to decrease the smoking rates within this population.

Moderate associations were present between age and employment status with Chinese immigrant smokers’ current smoking behavior. Chinese immigrants who were aged between 50 to 65 years old and unemployed tended to be current smokers. The association may be partially explained by a low-income level among this population, but further research needs to be carried out to understand the reasons for associations between employment status and current smoking behavior. Smoking cessation interventions that focus on elderly Chinese immigrants, especially the 50 to 65-year-old age group, and those unemployed, should be developed and implemented. Being elderly and smoking actively puts this population at a high risk of getting lung cancer. A low dose computed tomography screening and smoking cessation consultation are necessary health care interventions that need to be discussed in this population.

No significant associations were found among the variables measuring other demographic factors, psychological distress, healthcare utilization, and the current smoking status. This result was consistent with the findings in Samet et al.’s [25] study, which reported that languages spoken by physicians had no effect on smokers’ cigarette smoking status [25]. A lack of association between Chinese immigrants’ healthcare utilization and current smoking status may be caused by smokers’ and physicians’ indifference towards smoking cessation [27]. From Chinese immigrant smokers’ perspectives, they may not tend to view the physicians as someone to go to for advice to quit smoking, since they may believe a physician’s role predominantly is giving advice and treatment for illnesses as it is in other populations [28]. From the physicians’ point of view, health care providers may be reluctant to raise the topic of smoking cessation with their Chinese immigrant smoker clients during consultations, as they may fear this could cause confrontation with patients [28]. Thus, an ineffective smoking cessation consultation may lead to the healthcare utilization failing to change Chinese immigrant smokers’ current smoking behavior. In addition, psychological distress was found to not be associated with Chinese immigrants’ current smoking behaviors. This result is echoed by the findings from another study, which showed that psychological distress was related to smoking status for White but not for Black or Hispanic respondents [29]. Their results suggest that the often-reported association between psychological distress and smoking was relatively specific to White individuals, which may be caused by the faster metabolism of nicotine and severe nicotine withdrawal symptoms in this population [29]. Finally, gender, years lived in the US, level of English proficiency and spoken English use were found to not be associated with the current smoking behaviors among Chinese immigrants. Reasons for a non-significant relationship between gender and current smoking status in this study may attribute to the sample bias. Since this study consisted of a sample of people most of whom had a BA or BS degree or had some graduate school educational level, the possibility of a high smoking prevalence among male participants who had a low educational level was largely decreased.

### 4.1. Limitations

This study has some limitations. In the survey, the number of Chinese immigrants sampled was relatively small compared to the whole Chine immigrant group. However, adjusting for the survey weights allowed for generalization to the larger Chinese community being represented in California. Since California has the second-largest population of Chinese Americans [30], data collected in the survey was representative enough to represent Chinese immigrants in the United States. In addition, policies for tobacco use in California are different than those in other states. This may impact the smoking prevalence among Chinese immigrants in different geographic locations. However, this study still provided valuable evidence that could be used for comparing smoking prevalence in different areas. Furthermore, this study analyzed data from the 2016 CHIS. Although 2016 CHIS data are not up-to-date, relationships among demographic factors, psychological distress, healthcare utilization and current smoking behaviors among Chinese immigrants are relatively stable, or with the time as a third variable, it can still provide important information about the changes of the relationships. Lastly, this study used a cross-sectional design to explore the relationship between the demographic factors, psychological distress, healthcare utilization factors and current smoking status. A cross-sectional design cannot lead to the cause-and-effect results. We cannot draw conclusion from the study about what factors predict the smoking behaviors.

### 4.2. Implications for Practice and Research

Findings from this study could help healthcare educators and providers to provide culturally sensitive smoking cessation educations to the targeted Chinese immigrants. Chinese immigrants who are 50–65 years old, male, have less than a bachelor’s degree education level and a lower income are more likely to be current smokers. Health education about smoking cessation should focus on this group in the population to further decrease the smoking rates in this population. In addition, guided by the Integrated Model of Behavioral Prediction, health education on smoking cessation among Chinese immigrant smokers should integrate considerations about smokers’ demographics and cultural beliefs toward smoking cessation. Future research exploring Chinese immigrant smokers’ cultural beliefs about smoking cessation is necessary. Furthermore, income was found to be associated with the current smoking status in this study. However, whether income can predict smoking cessation, or whether smoking cessation is only related to personal motivation regardless of the income level, is not clear, and may need to be further explored in future studies. Lastly, in addition to the explored factors in the study, the acculturation level was suggested by previous studies as a factor that is possibly associated with Chinese immigrants’ current smoking behavior. According to Shelley et al. [31], acculturation was negatively related to being an ever-smoker and remained significant (*p* < 0.05) after adjusting for demographic variables (age, gender, educational level, marital status, employment status, regular source of care, insurance status, etc.), among 712 surveyed Chinese Americans. Future studies focusing on exploring relationships between acculturation level and current smoking status in Chinese immigrants are needed.

## 5. Conclusions

This study investigated the relationships among variables measuring demographic factors, psychological distress, healthcare utilization, and current smoking behaviors among Chinese immigrants. The results show that income was significantly associated with current smoking status among Chinese immigrants. Culturally sensitive smoking cessation programs targeted at low-income Chinese immigrant smokers could help to decrease the smoking rate in this population. Interventions focusing on the price policies related to tobacco use could potentially impact Chinese immigrants’ current smoking behaviors. More research needs to be carried out to help Chinese immigrants quit smoking.

## Figures and Tables

**Table 1:** Demographic variables (*n* = 650).

Variables	Categories	Weighted Percent (95% CI)
Age (years)	18–50	64.02 (53.12–74.90)
	50–65	21.93 (11.38–32.48)
	65+	14.05 (6.62–21.48)
Gender	Male	47.02 (35.02–59.01)
	Female	52.98 (40.99–64.98)
Marital status	Married	64.08 (49.04–79.12)
	Other widow/separate/divorce/living w/partners	13.13 (3.99–22.27)
	Never married	22.79 (10.75–34.83)
Spoken English use	Speak only English	11.63 (3.71–19.55)
	Very well/well	59.42 (45.64–73.21)
	Not well/Not very well	28.95 (16.64–41.25)
Level of English proficiency	Very well/well	67.25 (53.35–81.14)
	Not well/Not at all	32.76 (18.86–46.65)
Years lived in the US	<5 years	17.84 (4.44–31.24)
	5–14 years	16.65 (5.88–27.43)
	15+ years	65.51 (52.51–78.50)
Educational attainment	Below bachelor’s degree	26.78 (18.24–35.32)
	BA or BS degree/some graduate school	48.02 (34.87–61.16)
	Above bachelor’s degree	25.2 (14.60–35.81)
Employment status	Employed	69.2 (57.15–81.25)
	Unemployed	30.8 (18.75–42.85)
Income ^⊥^	weighted median (95% CI): 2451.63 (808.23–4095.04)	

^⊥^ Measured by the earnings in the last month.

**Table 2:** (**A**) Demographics by current smoking status among Chinese immigrants. (**B**) Income for previous month by smoking status among Chinese immigrants.

A			
Variables	Categories	Weighted Percentage (95% CI)	
Age (y)	18–50	0.53% (0–1.61%)	
	50–65	15.17% (0–35.20%)	
	65+	4.02% (0–13.83%)	
Gender	Male	5.95% (0–14.92%)	
	Female	2.70% (0–9.03%)	
Educational attainment	Below bachelor’s degree	12.80% (0–29.97%)	
	BA or BS degree/some graduate school	0.62% (0–2%)	
	Above bachelor’s degree	2% (0–7.21%)	
**B**			
**Variables**	**Categories**	**Weighted Mean (95% CI)**	**Weighted Median (95% CI)**
Income	Current smokers	$221.51 (−224.67–667.68)	$0 (−5013.46–5013.46)
	Not current smokers	$4339.55 (3007–5672.11)	$2566.28 (−594.29–5726.84)

**Table 3:** Weighted logistic regressions with current smoking as the outcome.

Outcomes	Covariates	*p*-Value
Demographic factors	Age	0.0565
	Gender	0.7673
	Marital status	0.7566
	Income	0.0471 *
	Employment status	0.0943
	Educational attainment	0.1322
	Spoken English use	0.7629
	Level of English proficiency	0.9118
	Years lived in the US	0.4840
Psychological distress	Distress	0.5744
Healthcare utilization	Personal doctor as a main medical provider	0.3794
	Language the doctor speaks	0.8425

* Significant result at *p* < 0.05.

## Data Availability

All the data analyzed in this study are available on the CHIS website: https://healthpolicy.ucla.edu/chis/ (accessed on 20 March 2023).
